# Case report: Emphysematous pyelonephritis associated with kidney allograft abscess formation

**DOI:** 10.3389/fmed.2022.1066512

**Published:** 2022-12-22

**Authors:** Bassam G. Abu Jawdeh, Michelle C. Nguyen, Margaret S. Ryan, Holenarasipur R. Vikram

**Affiliations:** ^1^Division of Nephrology, Mayo Clinic Arizona, Phoenix, AZ, United States; ^2^Division of Transplant Surgery, Mayo Clinic Arizona, Phoenix, AZ, United States; ^3^Department of Pathology, Mayo Clinic Arizona, Phoenix, AZ, United States; ^4^Division of Infectious Diseases, Mayo Clinic Arizona, Phoenix, AZ, United States

**Keywords:** immunosuppression, kidney, emphysematous pyelonephritis, kidney transplant recipient, graft nephrectomy

## Abstract

Emphysematous pyelonephritis (EPN) is a severe, acute necrotizing infection that is defined by the presence of gas in the kidney parenchyma. Multiple case reports have described the radiological findings and clinical course of EPN. Herein, we report on EPN including the histopathological findings in a kidney transplant recipient. Our patient presented with EPN complicated by multiorgan failure and was successfully managed with transplant nephrectomy.

## Introduction

Emphysematous pyelonephritis (EPN) is a rare, acute necrotizing kidney infection that is associated with high mortality (1, 2). Immunosuppression and diabetes mellitus are among the major risk factors predisposing to EPN. Herein, we describe a case of a diabetic kidney transplant recipient who presented with EPN associated with extensive cortico-medullary abscess formation requiring transplant nephrectomy.

## Case presentation

Our patient is a 49-year-old Caucasian woman who underwent deceased donor kidney transplantation in 2014 for end-stage kidney disease secondary to biopsy-proven diabetic nephropathy. Her medical history is significant for poorly controlled type 2 diabetes mellitus, with her most recent hemoglobin A1c being 11.5%, early post-transplant cytomegalovirus viremia, recurrent urinary tract infections, and mixed rejection (acute cellular rejection Banff 1B and antibody-mediated rejection) in the setting of donor-specific antibodies against human leukocyte antigen (HLA)-DR53 and HLA-DQ5 in 2019. The patient’s rejection episode was attributed to non-adherence with her mycophenolate/tacrolimus regimen suggested by subtherapeutic tacrolimus trough levels. At that time, she was treated with thymoglobulin, intravenous immunoglobulin, and plasmapheresis; however, she sustained chronic allograft insufficiency corresponding to chronic kidney disease stage 4.

She was transferred to our hospital in March 2022 with acute on chronic allograft injury, oligoanuria, abdominal pain, and delirium. At the time of transfer, she was afebrile, her blood pressure was low at 98/59 mm Hg, and her heart rate was 87 beats per minute. Lab results showed serum creatinine of 4.87 mg/dl (eGFR < 15 ml/min/1.73 m^2^), white blood cell count of 28,000 cells per cubic millimeter (neutrophil count, 27,000 cells per cubic millimeter), bicarbonate of 15 mmol/L, and anion gap of 16. The patient’s urinalysis revealed leukocyturia and microscopic hematuria, and the urine culture grew *Escherichia coli*. The blood cultures were negative.

**FIGURE 1:**
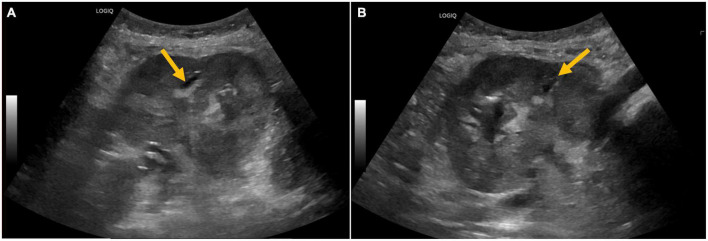
**(A,B)** Ultrasound of the kidney allograft with abnormal echogenicity and ill-defined hyperechoic areas with poor acoustic shadowing, raising suspicion of air in the pelvicalyceal system.

**FIGURE 2:**
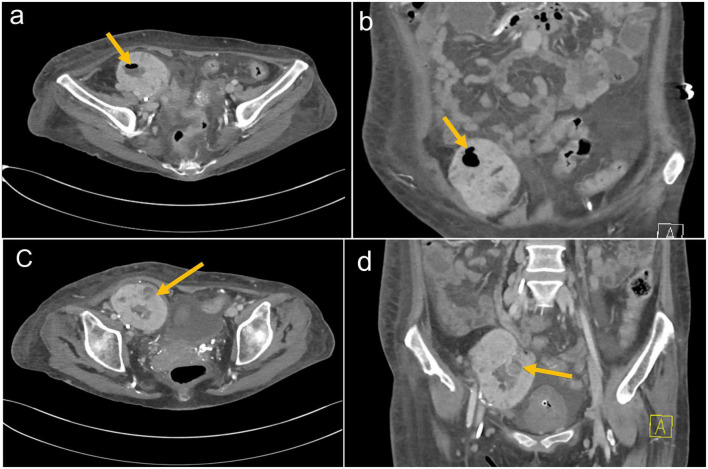
**(a)** Axial and **(b)** coronal CT images of the abdomen demonstrating the right lower quadrant kidney transplant with air in the renal collecting system. **(c)** Axial and **(d)** coronal CT images of the abdomen demonstrating right lower quadrant kidney transplant with loss of cortico-medullary differentiation and developing a complex intraparenchymal fluid collection with adjacent soft tissue perinephric rind. There is debris throughout the collecting system.

**FIGURE 3:**
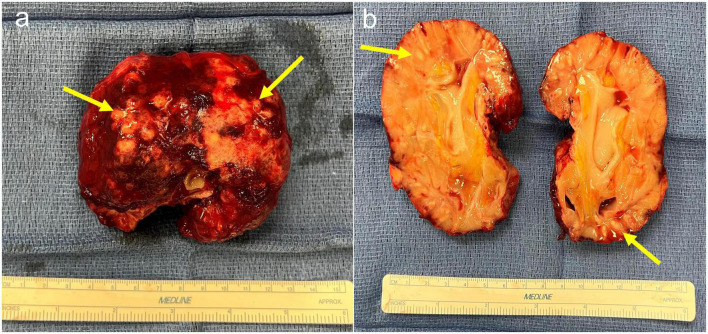
**(a)** Explant of kidney allograft demonstrating multiple abscesses with fibrinous exudate and thick rind material. **(b)** Bisected kidney allograft demonstrating diffusely pale parenchyma and multifocal areas of purulent exudate.

**FIGURE 4:**
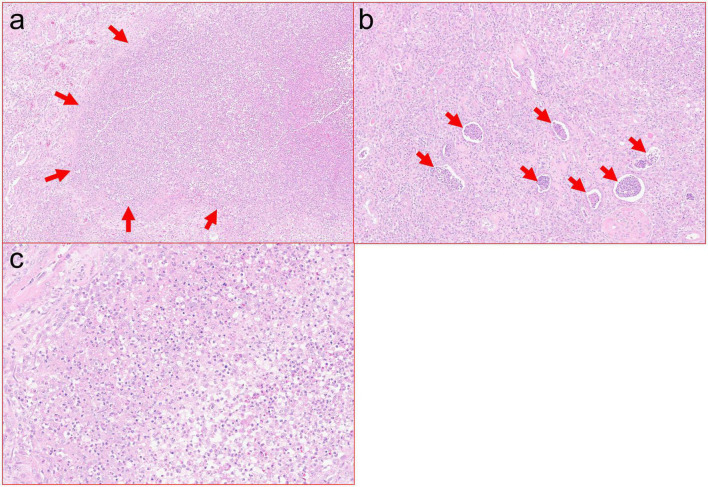
**(a)** Cortical abscess with marked neutrophilic inflammation and associated tissue destruction. Zones of tissue necrosis with abscess, such as pictured here, were present extensively throughout the resection specimen (H&E, 10×). **(b)** Severe interstitial neutrophilic inflammation with associated abundant intratubular abscesses (H&E, 20×). **(c)** High power view of neutrophilic inflammation with associated cellular debris (H&E, 40×).

Ultrasound of the right lower quadrant allograft was suspicious of intraparenchymal gas without any hydronephrosis (Figures 1A, B). CT scan of the abdomen showed EPN involving the transplanted kidney with possible perinephric abscesses and thickening of the urinary bladder consistent with cystitis (Figures 2a–d). The patient was initiated on vancomycin, ertapenem, and caspofungin, which were later de-escalated to ampicillin-sulbactam. She received fluids and insulin for sepsis and diabetic ketoacidosis. The patient’s acute kidney injury episode required initiation of hemodialysis, which was performed with a left internal jugular tunneled dialysis catheter. Based on ongoing abdominal pain and the patient’s deteriorating clinical course, a transplant nephrectomy was performed 4 days after admission (Figures 3a, b). Kidney allograft tissue culture grew *E. coli* with an antibiogram similar to the urine culture. Pathology of the explant showed extensive cortical and medullary abscess formation with associated tissue destruction (Figures 4a–c). There was no evidence of acute cellular or antibody-mediated rejection. The patient’s symptoms and leukocytosis subsequently improved. Her immunosuppression was tapered off over one week. She was discharged home 16 days after admission in stable condition and on chronic hemodialysis.

## Discussion

Emphysematous pyelonephritis is an acute necrotizing infection of the kidney that is associated with a significant mortality rate of up to 42% (3). EPN is defined by the presence of gas in the kidney parenchyma and/or collecting system (1). The gas is a result of glucose fermentation by Gram-negative bacteria, mainly *E. coli* (56%) and *Klebsiella pneumonia* (22%), which leads to the accumulation of carbon dioxide in the kidney tissue (4, 5). As a result, EPN is strongly associated with diabetes, with more than 80% of episodes occurring in diabetics (6). In addition to hyperglycemia, risk factors for EPN include immunosuppression, female gender, and urinary tract obstruction, most of which were present in our patient (5).

Al-Geizawi et al. proposed a three-stage classification system of EPN specific to kidney allografts (7). According to their classification, gas in the collecting system is stage 1, gas replacing <50% of kidney parenchyma and with well-controlled sepsis represents stage 2, whereas gas replacing >50% of parenchyma with extensive spread to the perinephric area or multiple organ failure corresponds to stage 3 (7). According to this classification, our patient fits stage 3, given her multiple organ failure, including acute kidney injury requiring initiation of dialysis and significant mental status changes. Indeed, aligning with our management plan, a recent review reported 100% of patients with stage 3 EPN received definitive treatment with transplant nephrectomy. This contrasts with one out of four and two out of nine patients with stages 1 and 2 EPN, respectively, undergoing transplant nephrectomy (4).

Multiple case reports have been published on EPN, mostly with an emphasis on imaging abnormalities. Here, we also report on the histopathological changes in the explant (Figures 3a, b, 4a–c). EPN in our patient was associated with significant neutrophilic inflammation, abscess formation, and tissue destruction leading to an acute deterioration of kidney function requiring dialysis. She had most of the risk factors, including female gender, diabetes, and immunosuppression.

## Conclusion

In conclusion, EPN should be considered in patients with poorly controlled diabetes presenting with sepsis, shock, or pyelonephritis. Underlying history of urinary obstruction and/or immune suppression, such as organ transplantation, can be an independent risk factor or further augment the EPN risk posed by uncontrolled diabetes. Prompt initiation of broad-spectrum antimicrobials, urgent abdominal imaging, and surgical consultation for native or allograft nephrectomy can be lifesaving.

## Data availability statement

The original contributions presented in this study are included in the article/supplementary material, further inquiries can be directed to the corresponding author.

## Ethics statement

Written informed consent was obtained from the patients OR patients legal guardian/next of kin for the publication of any potentially identifiable images or data included in this article.

## Author contributions

BA contributed to overseeing the work, drafting, and finalizing the manuscript and all the figures. MN contributed to drafting the manuscript and worked on the diagnostic radiology figures and captions. MR contributed to drafting the manuscript and worked on the pathology figures and captions. HV contributed to drafting the manuscript and providing critical revision of its content. All authors contributed to the article and approved the submitted version.
